# Diagnosing Mitochondrial Disorders Remains Challenging in the Omics Era

**DOI:** 10.1212/NXG.0000000000000597

**Published:** 2021-05-25

**Authors:** Patrick Forny, Emma Footitt, James E. Davison, Amanda Lam, Cathy E. Woodward, Spyros Batzios, Sanjay Bhate, Anupam Chakrapani, Maureen Cleary, Paul Gissen, Stephanie Grunewald, Jane A. Hurst, Richard Scott, Simon Heales, Thomas S. Jacques, Thomas Cullup, Shamima Rahman

**Affiliations:** From the Mitochondrial Research Group (P.F., S.R.), UCL Great Ormond Street Institute of Child Health; Metabolic Medicine Department (P.F., E.F., J.E.D., S. Batzios, A.C., M.C., P.G., S.G., S.R.), Great Ormond Street Hospital for Children NHS Foundation Trust; Neurometabolic Unit (A.L., S.H.), National Hospital for Neurology and Neurosurgery; Department of Chemical Pathology (A.L., S.H.), Great Ormond Street Hospital for Children NHS Foundation Trust; Neurogenetics Unit (C.E.W.), National Hospital for Neurology and Neurosurgery; Department of Neurology (S. Bhate), Department of Clinical Genetics (J.A.H., R.S.), North East Thames Regional Genetics Service, DBC Programme (T.S.J.), UCL Great Ormond Street Institute of Child Health and Department of Histopathology, and London North Genomic Laboratory Hub (T.C.), Great Ormond Street Hospital for Children NHS Foundation Trust, United Kingdom.

## Abstract

**Objective:**

We hypothesized that novel investigative pathways are needed to decrease diagnostic odysseys in pediatric mitochondrial disease and sought to determine the utility of clinical exome sequencing in a large cohort with suspected mitochondrial disease and to explore whether any of the traditional indicators of mitochondrial disease predict a confirmed genetic diagnosis.

**Methods:**

We investigated a cohort of 85 pediatric patients using clinical exome sequencing and compared the results with the outcome of traditional diagnostic tests, including biochemical testing of routine parameters (lactate, alanine, and proline), neuroimaging, and muscle biopsy with histology and respiratory chain enzyme activity studies.

**Results:**

We established a genetic diagnosis in 36.5% of the cohort and report 20 novel disease-causing variants (1 mitochondrial DNA). Counterintuitively, routine biochemical markers were more predictive of mitochondrial disease than more invasive and elaborate muscle studies.

**Conclusions:**

We propose using biochemical markers to support the clinical suspicion of mitochondrial disease and then apply first-line clinical exome sequencing to identify a definite diagnosis. Muscle biopsy studies should only be used in clinically urgent situations or to confirm an inconclusive genetic result.

**Classification of Evidence:**

This is a Class II diagnostic accuracy study showing that the combination of CSF and plasma biochemical tests plus neuroimaging could predict the presence or absence of exome sequencing confirmed mitochondrial disorders.

Mitochondrial diseases are multisystemic, complex disorders, which are difficult to diagnose. They are caused by pathogenic variants in at least 350 different genes across 2 genomes,^1^ and this list continues to grow with the frequent discovery of further disease genes. The challenge of finding a diagnosis for patients affected by a mitochondrial disorder arises from the variability in disease presentation regarding symptoms and age at onset^2^ as well as the lack of consistent biochemical markers.^3^ Many of the clinical symptoms of mitochondrial disease, for example, muscular hypotonia and weakness, gut dysmotility, and neurologic impairment, are shared by a wide range of different disease processes and so are not useful to identify the specific defective gene in a patient.^4^ Similarly, blood biochemical markers such as elevated lactate and alanine are helpful to raise the suspicion of a mitochondrial disorder but are not specific enough to point to a particular gene defect.^3^ Moreover, these biomarkers may be normal in individuals with genetically confirmed mitochondrial disease.^5^

Basic biochemical measurements, including plasma lactate, alanine, and proline, are routinely used to assess the probability of the presence of a mitochondrial condition. Elevations of these biomarkers are all due to the reduced mitochondrial utilization of pyruvate,^6^ which can be converted to lactate by lactate dehydrogenase or transaminated to form alanine.^7^ Raised proline is either due to inhibition of proline oxidase by lactate^8^ or increased proline synthesis from glutamate secondary to perturbed oxidative phosphorylation.^9^ In addition to these biomarkers, muscle biopsy is traditionally used to aid the diagnosis of mitochondrial disease.^10-12^ As muscle is a high-energy-requiring organ, mitochondrial defects frequently manifest in muscle tissue even in the absence of significant clinical myopathy.^13^ Muscle tissue investigations (respiratory chain enzyme activity [RCEA] analysis and muscle light and electron microscopy) have limited ability to predict a mitochondrial disorder, since they do not display consistent abnormalities in all cases. Moreover, muscle biopsy is invasive and associated with a risk of metabolic decompensation during general anesthesia, particularly in young children with mitochondrial disease. Finally, secondary RCEA deficiencies and histologic changes may be observed in several nonprimary mitochondrial diseases.^14^

To address this lack of diagnostic accuracy, the omics era brought new technologies, which are useful in assessing the genetic cause of a mitochondrial disorder at a wide and high-throughput scale.^15,16^ In this study, we used next-generation sequencing technology to investigate 85 consecutive patients with a suspected mitochondrial disorder in a single center by studying a clinical exome panel of 168 nuclear genes based on a shared gene panel developed by Genomics England,^17^ which includes moderate- and high-level evidence genes with regard to their disease association. Additional clinical exome panels with other disease foci were analyzed based on the clinical presentation of individual patients. The presented study describes the real-world experience of diagnosing mitochondrial disease using novel sequencing techniques and the associated challenges.

Overall, we aimed to compare traditional (hallmark clinical symptoms, basic biochemistry, and muscle tissue investigations) with a new method (exome sequencing and analysis of a mitochondrial disease gene panel) to identify the most useful instrument for first-line assessment of patients with a suspected mitochondrial disease at initial presentation.

## Methods

The primary research question addressed by this study was the identification of nongenetic (clinical, radiologic, and biochemical) predictors of exome sequencing failing to identify a genetically confirmed mitochondrial disorder (classification of evidence: Class II).

### Clinical Exome Sequencing

Extracted DNA was subject to library preparation using Agilent SureSelect XT (Agilent Technologies, Santa Clara, CA) and enrichment with a clinical exome bait set. For samples tested before January 2019, the Agilent Focused Clinical Exome was used, with added custom regions. For samples tested from January 2019, the Agilent Custom Constitutional Panel was applied. Enriched libraries were sequencing on an Illumina NextSeq500 instrument.

Data were parsed through a bioinformatic pipeline developed in-house, comprising alignment (Burrows-Wheeler Aligner), variant calling (freebayes), and variant annotation (Alamut batch). Filtering was performed to remove common variants (at greater than 2% overall minor allele frequency in the Exome Aggregation Consortium database) and common false-positive calls.

Variants passing filter were limited to target regions (Consensus Coding Sequence exons with 20 bp flanking intronic regions) of a virtual panel of genes. The panel of genes used for analysis was iterated several times over the time course of investigation to reflect changes in the PanelApp (panelapp.genomicsengland.co.uk/) nuclear mitochondrial gene set and the coverage of the relevant genes in the 2 captures. See tables e-1, e-2, and e-3 (links.lww.com/NXG/A427) for gene lists. Patient with identifiers (IDs) 1–14 were tested with the gene panel in table e-1, IDs 15–71 with the panel in table e-2, and IDs 72–85 with the panel in table e-3. In addition to the nuclear mitochondrial gene list, several patients had additional phenotype-specific gene lists included in their clinical exome analysis, reflecting the broad differential in their clinical diagnosis.

Variants were classified according to the guidelines of the American College of Medical Genetics and Genomics and the Association for Molecular Pathology^18^ with additional Association for Clinical Genomic Science guidance. Variants deemed to be clinically actionable (pathogenic/likely pathogenic variants in keeping with expected inheritance patterns, and variants of uncertain clinical significance for which segregation studies could lead to reclassification as pathogenic/likely pathogenic) were confirmed by Sanger sequencing. The diagnosis of a genetic mitochondrial disease was made based on the results of clinical exome sequencing (performed in all patients) and mitochondrial DNA (mtDNA) studies (performed in 66% of patients). The cohort of patients was not filtered or selected in any way after the clinical suspicion of a mitochondrial disorder was raised.

### Clinical and Biochemical Data Collection

Patient information including phenotypic information, neuroimaging studies, and routine biochemical test results was extracted retrospectively from the electronic patient record at Great Ormond Street Hospital, London, UK. Specialized tests were performed to measure urine organic acids in urine semiquantitatively as previously described^19^ and a range of acylcarnitine species in dried blood spots in a quantitative manner: free carnitine, acetyl carnitine (C2), propionyl carnitine (C3), butyryl carnitine (C4), isovaleryl carnitine (C5), hexanoyl carnitine (C6), octanoyl carnitine (C8), tetradecenoyl carnitine (C14:1), and palmitoyl carnitine (C16).^20^

### Muscle Histology Studies

Light and electron microscopy were performed as previously described.^21^ Abnormal histologic findings included ragged red fibers and fibers staining negatively for cytochrome *c* oxidase (COX). Although nonspecific, findings such as excess lipid deposition, increased variation in fiber size, and abnormal morphology of mitochondria on electron microscopy were also classified as abnormal when they were present in large amounts. Mildly abnormal samples showed subtle changes, including little excess lipid, minimal variation in fiber size, and mild atrophy.

### Muscle Respiratory Enzyme Activity Assay

For assessment of RCEA, muscle was snap-frozen on collection, and spectrophotometric assays of respiratory chain complexes I to IV were performed according to previously reported methods.^22^

### Statistical Analysis

Statistical testing and figure design were facilitated by R version 4.0.0. Continuous and discrete variables were correlated with the outcome of a positive mitochondrial diagnosis on exome sequencing by applying logistic regression. Some variables were transformed to log scale before the analysis to resemble normal distribution (figure e-1, a and b, links.lww.com/NXG/A427). To estimate effect size, pseudo *R*^2^ was calculated according to the McFadden method. Receiver operating characteristic (ROC) curves were drawn by applying the R package plotROC (version 2.2.1), the correlogram by application of the ggcorrplot R package (version 0.1.3). Significance levels of *p* values are indicated as follows throughout the study: *p ≤ 0.05 and **p ≤ 0.01.

### Standard Protocol Approvals, Registrations, and Patient Consents

Verbal consent was obtained from all patients (or their legal guardians) involved in the study. This study was approved by an institutional committee and was registered as a clinical audit at Great Ormond Street Hospital NHS Foundation Trust (audit no. 2381). All ethical institutional requirements have been fulfilled and were followed during this retrospective study.

### Data Availability

Exome data cannot be supplied as they are patient specific. All novel variants detected in the study will be made publicly available on the ClinVar platform (ncbi.nlm.nih.gov/clinvar/).

## Results

### Clinical Patient Cohort Characterization

**Figure 1:**
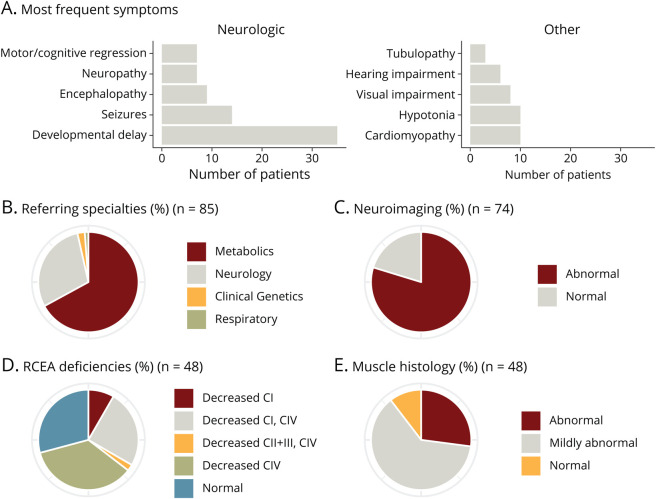
Patient Cohort Characterization (A) Bar chart depicting number of patients in whom a specific symptom was found. (B) Referral of patients for clinical exome sequencing per medical specialty. (C) Pie chart depicting proportions of general neuroimaging outcomes. (D) Summary of the outcome of respiratory chain enzyme activity (RCEA) measurements in muscle biopsy. (E) General muscle (light and electron) histology results. For all pie charts, n values indicate in how many patients the investigation was performed.

This is a retrospective review of 85 patients (44 females and 41 males) with suspected mitochondrial disease who were recruited consecutively for mitochondrial disease nuclear gene panel analysis in the Great Ormond Street Hospital Clinical Exome (GOSHome) from January 2015 to November 2018. The overall median age at presentation was 0.50 years (mean 1.92; SD 3.54) (figure e-2a, links.lww.com/NXG/A427). The most frequent clinical symptoms were neurologic and included global developmental delay (n = 35), seizures (n = 14), hypotonia (n = 10), and encephalopathy (n = 9); cardiomyopathy (n = 10) was also a frequent finding (figure 1A). The patients were recruited for exome sequencing from different pediatric disciplines in a tertiary clinical setting, including Metabolic Medicine (n = 57), Neurology (n = 25), Clinical Genetics (n = 2), and Respiratory (n = 1) (figure 1B). Seventy-four patients underwent neuroimaging by MRI, which showed abnormal findings in 80% (n = 59) of patients (figure 1C). In 53 patients (62% of all patients), muscle tissue was biopsied and investigated (skeletal muscle n = 52; cardiac muscle n = 1), of which 48 (5 not assayed due to delayed freezing) were tested for mitochondrial RCEA, namely complex I, II + III (assessed together), and IV. Individual patients either showed single or combined RCEA deficiencies, specifically isolated loss of complex I (n = 4), combined loss of complex I and complex IV (n = 12), combined loss complexes II + III and IV (n = 1), isolated loss of complex IV activity (n = 17), or no evidence of a mitochondrial RCEA deficiency (n = 14) (figure 1D, table e-4). Furthermore, in 48 of the patients who underwent muscle biopsy, histology investigations were performed and revealed normal (n = 5), mildly abnormal (n = 29), and abnormal (n = 14) findings (figure 1E).

### Mitochondrial Disease Genes

Molecular genetic analysis of patient samples was performed using a clinical exome panel. Genes encoded by mtDNA were investigated by other means (screening for common point mutations and large-scale rearrangements and/or complete mitochondrial genome sequencing, and mtDNA quantification) in 56 cases (66% of all patients). The 85 patients were referred for clinical exome sequencing at a median age of 5.92 years (mean 6.71, SD 5.09) (figure e-2a, links.lww.com/NXG/A427), which represents a considerable delay after their initial clinical presentation (time of delay: median 3.67 years, mean 4.79, range 0–16.50 years) (figure e-2b). The time from clinical presentation to completed clinical exome sequencing was a median of 4.16 years, showing a high variability (range 0.33–17.08 years) (figure e-2, b and c). This might have been partly attributable to a lack of clinical (i.e., nonresearch) diagnostic genome-wide genetic testing before the development and implementation of the GOSHome. The median time from request to report was 196 days, with some cases resolved within just over a month and others that took more than 1 year (range 48–418 days) (figure e-2b). The variance in reporting time can be explained by clinical urgency, with some cases prioritized to assist clinical management or reproductive options, and case complexity; patients in whom complex variants were identified that required additional validation assays took longer to reach reporting status.

Twenty-five of the 85 patients received a confirmed genetic diagnosis via the clinical exome panel, with the following affected genes: *FBXL4* (n = 4), *AARS2* (n = 2), *ELAC2* (n = 2), *LRPPRC* (n = 2), *C12orf65*, *CA5A*, *CACNA1A*, *EARS2*, *EXOSC3*, *FOXRED1*, *GRIN2A*, *IBA57*, *LAMA2*, *MMAB*, *NDUFB11*, *PDHA1*, *RMND1*, *SDHA*, and *VPS13B* (all n = 1; underlined genes are not considered mitochondrial disease genes and were identified via additional panel analysis) (table 1). One additional patient received a probable diagnosis with only 1 heterozygous mutation found in *ECHS1*. Although we have not identified a second *ECHS1* variant in this patient, enzymatic studies confirmed a deficiency of enoyl-CoA hydratase (ECHS1) (patient ID 75). Of note, some of the identified nuclear genes encode mitochondrial proteins but are not considered to cause a classical mitochondrial condition and were identified via alternative panels as part of the clinical exome sequencing analysis (*CA5A* in patient ID 32; *MMAB* in patient ID 36). In addition, 5 patients were diagnosed via alternative analyses. Three patients were diagnosed with mtDNA mutations by direct mitochondrial genome sequencing because the clinical exome sequence analysis pipeline applied in this study was unable to detect mtDNA mutations. Two cases had known pathogenic mtDNA variants: m.13514A>G p.(Asp393Gly) in *MT-ND5* (patient ID 40) and m.10197G>A p.(Ala47Thr) in *MT-ND3* (patient ID 49). For the third patient, we report a novel pathogenic variant m.3955G>A p.(Ala217Thr) in *MT-ND1* as a new cause of Leigh syndrome in a boy who presented at 2 months with an early infantile epileptic encephalopathy, lactic acidosis, and brain MRI demonstrating bilateral symmetrical signal abnormality involving the caudate nuclei, putamina, midbrain, medulla, upper cervical cord, and perirolandic cortex. The variant was heteroplasmic, with 89% mutation load in blood DNA from the affected infant but was absent in blood DNA from his unaffected mother. The variant has never previously been reported and is absent in MitoWheel and MITOMAP (mitowheel.org, mitomap.org; both accessed on October 17, 2020). Previously reported pathogenic variants in *MT-ND1* include m.3376G>A, p.(Glu24Lys) reported to cause a Leber hereditary optic neuropathy/mitochondrial encephalopathy, lactic acidosis, and stroke-like episodes (MELAS) overlap syndrome and m.3697G>A,p.(Gly131Ser), associated with MELAS and Leigh syndromes. In 2 cases, a diagnosis was established by whole-exome sequencing: patient ID 22 was found to be compound heterozygous for 2 mutations in *TBC1D24*: c.901C>T p.(Gln301Ter) and c.605C>T p.(Ser202Leu), and patient ID 34 carried the homozygous pathogenic mutation c.121A>T p.(Lys41Ter) in *NKX6-2* (table 1). The global diagnostic rate in our study was 36.5%. Overall, 24 patients were identified with a genetically confirmed mitochondrial disease, and we are reporting a total of 20 novel disease-causing variants (table 1). Seven patients were diagnosed with a nonmitochondrial disorder (table 1).

**Table 1:**
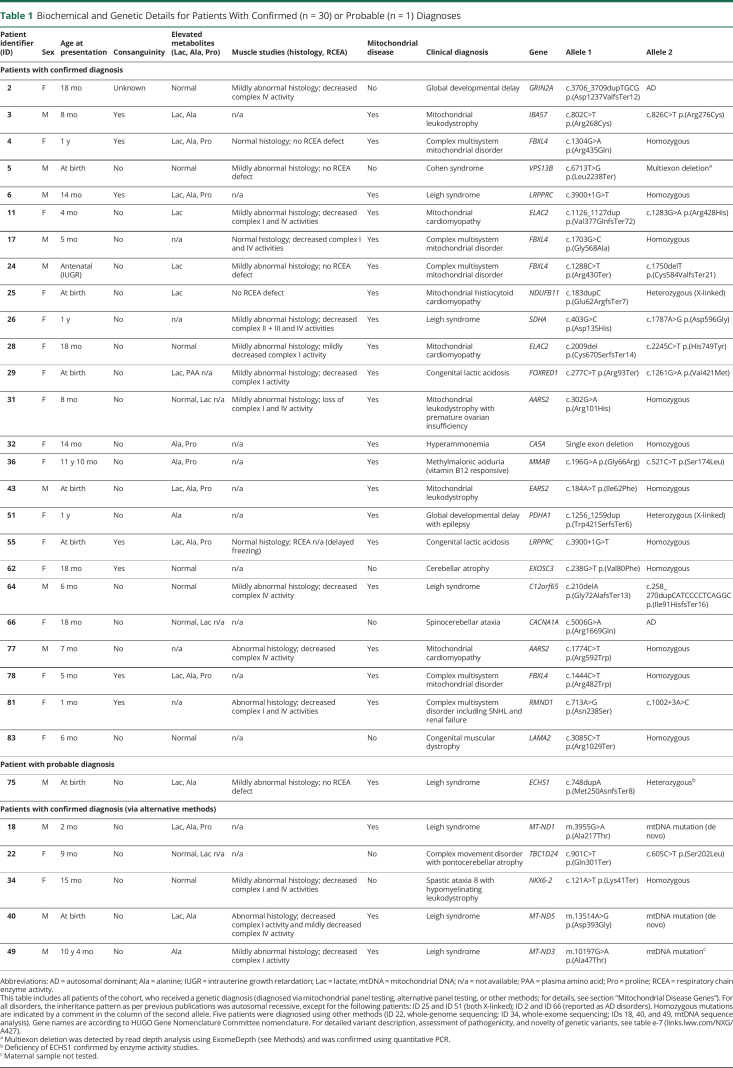
Biochemical and Genetic Details for Patients With Confirmed (n = 30) or Probable (n = 1) Diagnoses

### Biochemical Phenotyping

**Figure 2:**
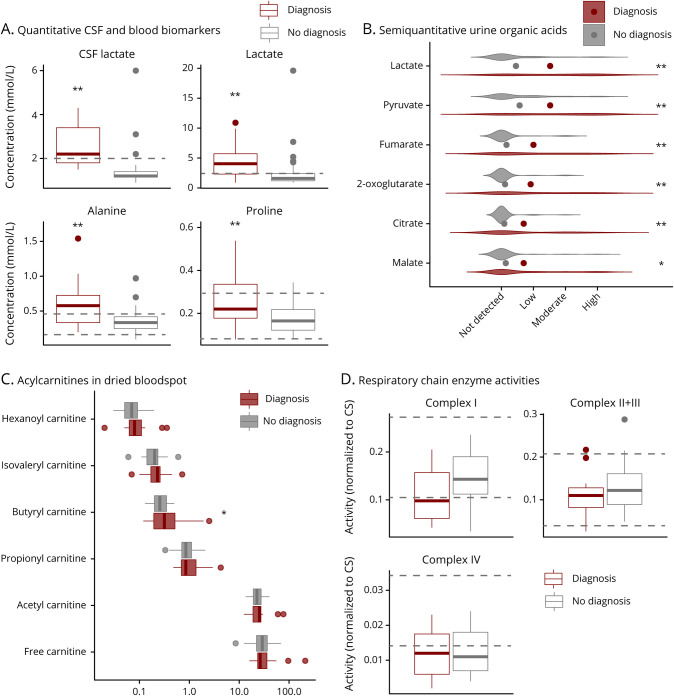
Biochemical Characterization (A) Boxplots of CSF and plasma biomarkers. Dashed horizontal lines indicate reference ranges; for CSF lactate and plasma lactate, normal values are below the dashed horizontal line. (B) Violin plots illustrating results of semiquantitative urine organic acid measurements, points represent mean values. (C) Horizontal boxplots of acylcarnitine results. Boxplots consist of horizontal marks for the 25th percentile, the median, and the 75th percentile, whiskers extend to the 5th and 95th percentile, and outliers are represented as separate individual values. (D) Boxplot of levels of respiratory chain enzyme activities normalized to citrate synthase (CS) activity. Dashed horizontal lines indicate reference ranges. All plots are split in 2 groups of patients with a mitochondrial diagnosis in red and those with no mitochondrial diagnosis in gray. Significance of differences was determined by logistic regression analysis. * = *p* ≤ 0.05; ** = *p* ≤ 0.01.

The patients had undergone a variety of different test modalities before genetic sequencing (figure e-3a, links.lww.com/NXG/A427). Routine biochemical investigations revealed various abnormalities, including elevated lactate in CSF (n = 9, as assessed in 36 patients, i.e., in 25% in whom the test was performed) and plasma (n = 23, as assessed in 68 patients, i.e., in 34%), elevated plasma alanine (n = 26, as assessed in 75 patients, i.e., in 35%), and plasma proline (n = 15, as assessed in 75 patients, i.e., in 20%) (figure e-3b). All 4 tests were able to distinguish between patients with mitochondrial disease and patients without a diagnosis of a mitochondrial disorder (figure 2A). Consistent with raised CSF and plasma lactate levels, semiquantitative urine organic acid analysis revealed elevated levels of urine lactate and pyruvate in the mitochondrial patient group (figure 2B). In addition, several metabolites of the Krebs cycle were also significantly elevated, such as fumarate, 2-oxoglutarate, and citrate (figure 2B). Quantification of acylcarnitine species showed a significant elevation of butyryl carnitine (figure 2C). Surprisingly, quantitative RCEA measurements showed no correlation with the presence of a confirmed mitochondrial disease diagnosis (figure 2D).

### Predictive Parameters of Mitochondrial Disease

**Figure 3:**
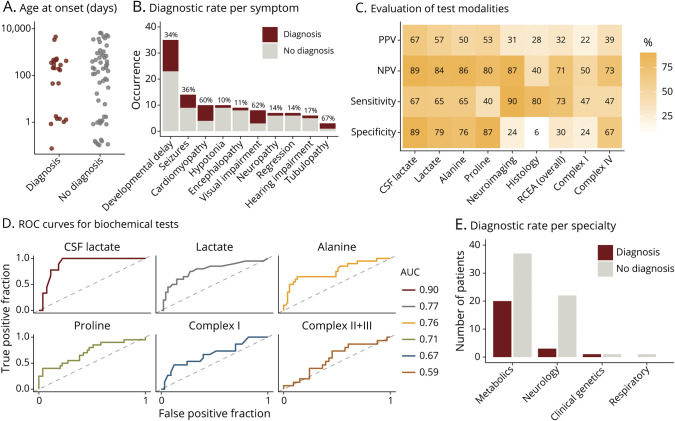
Predictive Parameters for Mitochondrial Disease The panels in this figure describe comparisons between patients with a mitochondrial diagnosis (red) and patients with no diagnosis (including nonmitochondrial diagnosis, gray). (A) Age at presentation of individual patients; for better visibility on the log scale (y-axis), patients with antenatal abnormalities were assigned an age at onset of 0.1 day and for onset at birth of 1 day. (B) Bar chart illustrating the occurrence of the most frequent symptoms in the cohort, including diagnostic rate in percent for each individual symptom above the bars, as calculated by number of patients with this symptom and a mitochondrial diagnosis divided by the number of all patients with this symptom. (C) Heatmap depicting levels of positive predictive value (PPV), negative predicate value (NPV), sensitivity, and specificity for different test modalities, calculated using a genetic mitochondrial diagnosis as the gold standard. In the calculations, a positive outcome of test modalities was considered as follows: raised levels of lactate, alanine, and proline, decreased activity of RCEA complexes, and abnormal neuroimaging, RCEA, or histology. (D) ROC curves for selected continuous test variables. (E) Bar graph depicting the outcome of clinical exome analysis per referring specialty. AUC = area under the ROC curve; RCEA = respiratory chain enzyme activity; ROC = receiver operating characteristic.

To identify predictive factors for the presence of a mitochondrial disorder, we investigated the correlation of clinical symptoms and investigation findings with a positive mitochondrial disease genetic diagnostic outcome, as obtained in 24 patients. Age at presentation did not correlate with a genetic diagnosis of mitochondrial disease (figure 3A). Of the wide range of clinical symptoms found in the cohort, most single symptoms were not strongly correlated with a positive diagnosis (figure 3B). The highest diagnostic rates were noted for cardiomyopathy (60%), visual impairment (62%), and renal tubulopathy (67%, only in n = 3 patients), whereas the 2 former symptoms were the only clinical symptoms significantly associated with a mitochondrial disease diagnosis (figure 3B). Of interest, a third (34%) of all patients with the nonspecific symptom of global developmental delay received a positive diagnosis (figure 3B). Other prevalent symptoms in this cohort were not predictive of a positive genetic diagnosis.

As the clinical assessment does not seem to be able to stratify between patients with mitochondrial disease and patients without mitochondrial disease in our cohort, additional test modalities were evaluated regarding their ability to predict mitochondrial disease (figure 3C). Elevated metabolites in CSF and plasma (lactate, alanine, proline) were consistently associated with a higher positive predictive value (50%–67%) compared with other tests (figure 3C). The same test modalities together with neuroimaging proved even more useful with regard to a high negative predictive value, which would allow exclusion of a mitochondrial disorder in the case of normal results in 80%–89% of cases (figure 3C). Elevated biomarkers (at least 1 out of lactate, alanine, or proline) showed an unexpectedly high specificity in our patient cohort, ranging from 76% to 89% (figure 3C). As expected from a biological perspective, these biomarkers correlated positively with each other (figure e-4, links.lww.com/NXG/A427). Of interest, although we observed a linear relationship between plasma lactate and the other 2 plasma metabolites in nonmitochondrial patients, the levels of alanine and proline were independent of lactate in mitochondrial disease patients (figure e-5). These biomarkers also perform well in terms of sensitivity (except for proline). Abnormal RCEA and abnormal muscle histology are similarly sensitive parameters (figure 3C). Although muscle tests showed a reasonable sensitivity, neuroimaging was the most sensitive test modality (90%) (figure 3C). When it comes to positive predictive value and specificity, RCEA testing and histology investigations performed poorly; abnormal RCEA only showed a positive predictive value of 32% (11/34) and abnormal muscle histology of 28% (12/43) (figures 3C and e-3b). Ragged red fibers and COX-negative fibers were specific for mitochondrial disease, as expected, but had low sensitivity, being detected in only 1 patient each (patient IDs 40 and 81, respectively), both of whom were diagnosed with a genetically confirmed mitochondrial disease (pathogenic variants in *MT-ND5* and *RMND1*, respectively). Other samples with a confirmed gene defect affecting mitochondrial translation (*AARS2*, *C12orf65*, *EARS2*, *ELAC2*, and *LRPPRC*) did not show any of these 2 mitochondrial hallmark histology findings. Additional histology findings such as excess lipids, variation in fiber size and type, or abnormal mitochondrial morphology did not correlate with a positive mitochondrial diagnosis (table e-5).

Further evaluation of diagnostic test modalities by ROC curve analysis revealed that CSF lactate is the best continuous variable in the data set to distinguish between a patient with mitochondrial disease vs a nonmitochondrial disease state with an area under the ROC curve of 0.91 (figure 3D). The aforementioned simple plasma metabolite tests (lactate, alanine, and proline) performed reasonably and again better than the more sophisticated studies of RCEA in muscle tissue (figure 3D). Other continuous parameters such as plasma arginine or age at presentation were not able to predict mitochondrial disease in the study cohort (figure e-6, links.lww.com/NXG/A427).

Of the referring specialties, only Neurology and Metabolic Medicine provided a large number of patient samples: the latter showed a significantly higher rate of positive mitochondrial diagnoses (35%) compared with the former (12%) (figure 3E), which may reflect the types of patients referred to each of these specialties.

**Figure 4:**
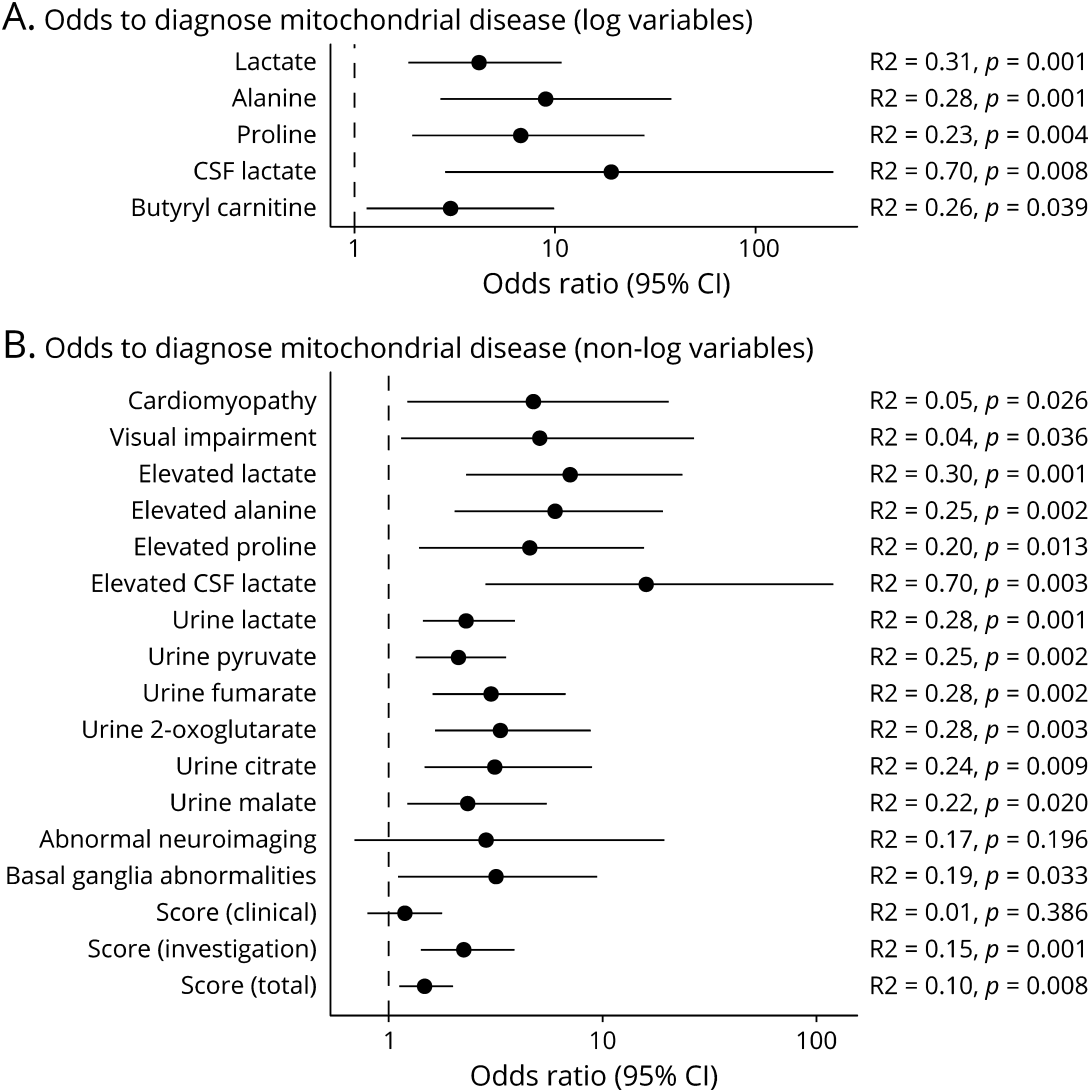
Odds Ratios of Predictive Parameters Forest plot depicting effect sizes of diagnostic measures, including 95% confidence interval (CI), derived from logistic regression analysis. (A) These continuous variables were log transformed before analysis. (B) These variables are all discrete, e.g., presence or absence of cardiomyopathy and presence or absence of elevated lactate. *R*^2^ = *R*^2^ according to the McFadden method.

To quantify the effect size of each test modality, we performed logistic regression analysis and calculated odds ratios for each continuous variable in the data set (tables e-5 and e-6, links.lww.com/NXG/A427). Depending on their distribution, specific continuous variables were log transformed before the analysis (figure e-1). In line with the previous statistical analysis, CSF lactate is a strong predictor of mitochondrial disease and increases the odds of a mitochondrial diagnosis by 16 times in case of an elevation above the reference range (figure 4, A and B). Previously mentioned tests, such as other lactate associated parameters as well as Krebs cycle metabolites, showed a significantly raised odds ratio (figure 4, A and B). Of interest, only bilateral basal ganglia abnormalities were associated with a mitochondrial disease diagnosis in our study cohort, whereas other neuroimaging findings including cerebellar hypoplasia, cerebral atrophy, and white matter abnormalities were not (figure 4B, table e-5).

### Mitochondrial Disease Score

The suspicion of a mitochondrial disorder for each patient in this study was not based on a systematic assessment. The clinical exome investigation was initiated based on the individual clinician's judgment. We have retrospectively applied a mitochondrial disease score developed in this study, which was based on a previous mitochondrial disease score.^23^ The presence of one of the 10 most prevalent symptoms in the overall cohort (figure 1A) scored 1 point each (maximum clinical subscore of 10 points), whereas abnormal neuroimaging, biochemistry (lactate, alanine, proline, and RCEA; 1 point each), and muscle histology scored 1 point each (maximum investigation subscore of 6 points), representing clinical symptoms as well as biochemical/imaging aberrations. When the score was applied to the patient cohort of this study, it ranged from 0 to 9 (figure e-7a, links.lww.com/NXG/A427), and the mean overall score of the 85 patients was 3.64 (SD 1.85) (figure e-7b). The overall score and the investigation subscore were significantly associated with a positive mitochondrial diagnosis (figures 4B and e-7b), and a total score >4 points should trigger genetic follow-up investigations in a patient with suspected mitochondrial disease.

## Discussion

In this study, we used clinical exome sequencing to search for a genetic diagnosis in patients with clinically suspected mitochondrial disease and compared the results with traditional markers. A genetic diagnostic confirmation was established in over a third of all cases. For the remaining patients, the question remains as to whether they had a mitochondrial disorder at all or whether their phenotype only mimicked this group of conditions.^4^

The limited diagnostic rate can be partly explained by the method of exome sequencing applied in this study, which might have missed some of the disease-causing variants due to several limitations, including (1) limited number of genes in the clinical panel; (2) analysis of mtDNA in only 66% of patients; (3) potential for insufficient coverage of certain regions; (4) test sensitivity for certain variant types—copy number testing was performed as standard using ExomeDepth, but the sensitivity is unknown, and the sensitivity to detect insertion/deletion variants using short read sequencing is also known to be low when compared with single nucleotide variants; and (5) causative variants lying outside the target region (e.g., promoter, untranslated regions, and deep intronic variants). Addressing the above points in the undiagnosed participants of our study might yield more diagnoses of mitochondrial disease, although generally speaking, mtDNA analysis was not performed in those patients deemed to have a lower suspicion of mitochondrial disease. Although there are some drawbacks of the method applied in this study, the application of a panel-based clinical exome sequencing method also has some clear advantages compared with whole-exome or genome approaches, e.g., the number of variants of unknown significance is lower because only a subset of genes is specifically analyzed. Some variants, especially if previously published in relation to a distinct disease phenotype, are classified as pathogenic, but the remaining variants are often difficult to interpret. Therefore, we emphasize that clinical exome sequencing should never be used as an isolated method, but always accompanied by a set of follow-up procedures, including segregation studies in the family, complementary DNA sequencing if indicated, and most importantly functional testing where necessary (e.g., as in patient ID 75 in this study).

When comparing the outcome of the clinical exome analysis in our study with predictive parameters, our study demonstrates that single clinical symptoms or the application of a clinical score are overall poor predictors of mitochondrial disease, as previously discussed.^4^ However, the combination of suspicious clinical features with the results of noninvasive methods such as neuroimaging (excellent sensitivity) and biomarkers (in CSF, blood, and urine) such as lactate, pyruvate, and alanine (high specificity) can significantly improve the pretest probability of a mitochondrial disorder. These basic investigations should therefore be completed before clinical exome sequencing is initiated, depending on the clinical urgency, potentially together with other promising biomarkers currently being established in the field, such as FGF21 and GDF15.^24^ Similar strategies are described in current guidelines for diagnosis and management of mitochondrial disorders,^6,23^ and comparable rates of sensitivity and specificity for biomarkers have been described.^25,26^

Furthermore, we have compared the results of muscle investigations performed in the majority of our patients with the outcome of the clinical exome analysis. Previous exome studies for mitochondrial disease have not performed such a direct comparison of exome and muscle investigations. Thus, our parallel clinical exome and muscle data set will provide an important historical resource going forward, as fewer muscle biopsies are performed for mitochondrial disease worldwide. Our data reveal that the traditional muscle techniques have a poor diagnostic specificity, and it could be argued that muscle biopsies should be avoided in the future as a first-line test, and instead, clinical exome analysis should be performed as a priority when the suspicion of a mitochondrial disorder has been raised during the initial clinical and biochemical assessment of the patient. Even in clinically urgent situations, such as the neonatal/pediatric intensive care unit, next-generation sequencing methods might yield an earlier result than investigation of muscle tissue, as shown for Pearson syndrome and other conditions.^27,28^ Our study shows that muscle histology was completely normal in only 10% of all cases, which were analyzed with this technique. The insufficient specificity was further underlined by the result of more than 60% of cases with slightly abnormal histology, precluding a definite diagnosis based on muscle histology. Furthermore, RCEA measurements in muscle show a low positive predictive value and were not specific enough to reliably diagnose mitochondrial disease. These conclusions are limited by the fact that not all patients in this cohort were investigated by mtDNA analysis.

On the basis of these results, we suggest that analyses relying on muscle biopsy material should be replaced by clinical exome sequencing in the initial stages of the mitochondrial diagnostic workflow. Muscle biopsy still has a role in the evaluation of suspected mitochondrial disease and should be considered in the following 3 scenarios: (1) critical illness; (2) for confirmation of pathogenicity of variants of unknown significance identified by exome or genome sequencing; and (3) if genetic investigations do not yield any positive findings and a strong clinical suspicion of mitochondrial disorder remains.

Finally, the initial contact between the physician and the patient will remain the crucial key step in finding a diagnosis for patients affected by a mitochondrial disease, and there is no substitute for thorough history taking and physical examination.^2^ Our study illustrates that numerous patients go through a years-long diagnostic odyssey. We can only speculate on the reasons for the diagnostic delay illuminated by our data: (1) access to diagnostic methods: the clinical exome analysis developed in this study could have presented the first opportunity to the referring physician to actually perform a wider genetic analysis; and (2) uncertainty of clinical course: our study shows that in younger patients, the diagnostic delay is particularly long, possibly reflecting a wait-and-see approach in younger children with potentially nonspecific clinical signs. We expect that costs for exome studies will continue to fall^29^ and turnaround times will further improve, enabling early recruitment of patients for exome or genome-wide studies and consequently shortening the diagnostic odyssey for patients with suspected mitochondrial disease.

## Glossary

COX: cytochrome *c* oxidase
GOSHome: Great Ormond Street Hospital Clinical Exome
ID: identifier
MELAS: mitochondrial encephalopathy, lactic acidosis, and stroke-like episodes
mtDNA: mitochondrial DNA
RCEA: respiratory chain enzyme activity
ROC: receiver operating characteristic
